# Missing data, missed risks: complications and documentation gaps of central venous access devices in pediatric oncology

**DOI:** 10.1186/s12887-026-06612-0

**Published:** 2026-02-10

**Authors:** Marie Voigt, Alexandros Rahn, Anna-Lena Herbach, Urs Mücke

**Affiliations:** 1 Department of Pediatric Hematology and Oncology, Hannover Medical School, Carl-Neuberg-Str. 1, 30625 Hannover, Germany; 2 Department of Pediatric Pulmonology, Allergology and Neonatology, Hannover Medical School, Carl- Neuberg-Str. 1, Hannover, 30625 Germany

**Keywords:** Central venous access device, Pediatric oncology, Catheter-related complications, Infection, eDelphi, Surveillance

## Abstract

**Background:**

Central venous access devices (CVADs) are essential in pediatric oncology but frequently associated with infections, thromboses, and dysfunction. Although structured surveillance may reduce complications, implementation is limited by fragmented documentation across clinical systems.

**Methods:**

We conducted a mixed-methods study combining a two-stage interdisciplinary electronic Delphi process with retrospective cohort analysis. Pediatric oncology patients diagnosed between 2020 and 2021 who received a CVAD at our university hospital were included. Data were manually extracted from three hospital information systems. Only the first CVAD per patient was analyzed (*n* = 112).

**Results:**

Of 219 diagnosed patients, 112 met inclusion criteria, accounting for 126 CVADs. The eDelphi panel identified 30 core surveillance parameters, 23 retrievable from routine records. Complications occurred in 23.2%, most commonly infections leading to premature removal.

**Conclusions:**

Standardized electronic CVAD documentation could enhance surveillance, improve patient safety, and reduce manual data collection efforts.

**Supplementary Information:**

The online version contains supplementary material available at 10.1186/s12887-026-06612-0.

## Background

Pediatric cancer requires intensive multimodal therapies that rely on dependable long-term venous access [1]. Although malignant diseases in childhood are relatively rare, approximately 2,300 new cases are diagnosed annually in Germany, with leukemias, central nervous system tumors, and lymphomas representing the most common entities [2]. Advances in diagnostics, treatment strategies, and supportive care have substantially improved survival over recent decades, yet treatment-related complications remain a major contributor to morbidity and unplanned hospitalizations [3]. Reliable vascular access is therefore an essential component in pediatric oncology patients requiring intensive intravenous therapy. Central venous access devices (CVADs) enable the administration of chemotherapy, transfusions, parenteral nutrition, and repeated blood sampling throughout prolonged treatment courses [4]. Totally implanted venous access devices (TIVADs, ports) and tunneled cuffed central venous catheters (TC-CVCs) are the most frequently used CVADs in pediatric oncology. TIVADs consist of a subcutaneously implanted reservoir connected to a central venous catheter, whereas TC-CVCs are external catheters placed through a subcutaneous tunnel and secured by a cuff, allowing continuous access. These systems differ in invasiveness, handling, and complication profiles [5, 6]. Despite their routine use, CVADs are associated with clinically relevant adverse events, including bloodstream infections, thrombosis, dislocation, and obstruction. Reported complication rates approach 20% in pediatric oncology cohorts, and device-associated bloodstream infections represent a major source of morbidity, delays in therapy, and increased healthcare costs [3, 7, 8].

Effective surveillance is essential to reduce CVAD-related complications [9, 10]. However, standardized prospective monitoring in pediatric oncology remains limited. Documentation practices vary widely across institutions and even between departments within the same hospital [11]. Data relevant to CVAD use, such as device characteristics, access procedures, complications, and microbiological results, are often distributed across multiple clinical information systems (CIS) [12]. This fragmentation hinders routine surveillance, inflates the manual workload required for retrospective analyses, and limits comparability across centers. As a result, true complication rates may be underestimated, and opportunities for quality improvement remain unrealized [9, 11].

International recommendations and standardized datasets for vascular access surveillance have been published, including the international vascular access minimum dataset derived through a modified Delphi process [12]. These frameworks provide an essential foundation for standardized reporting and benchmarking but are primarily designed for research and registry settings and are not specifically tailored to the documentation workflows and clinical context of pediatric oncology. Moreover, they do not systematically address the feasibility of capturing such parameters within heterogeneous routine clinical documentation systems. To address this gap, a structured and interdisciplinary operationalization of surveillance parameters is needed, alongside an evaluation of how well these parameters are currently documented in routine care. Establishing such a foundation could enable future prospective monitoring, facilitate benchmarking, and support the development of targeted interventions aimed at improving patient safety.

The primary aim of this study was to identify clinically relevant parameters for structured CVAD surveillance through an interdisciplinary electronic Delphi process (eDelphi) at a tertiary university hospital in northern Germany. As a secondary objective, we performed a retrospective chart review to characterize the study population, CVAD use, and documented complications, with a particular focus on infectious events. In addition, the chart review was used to illustrate the availability and quality of selected consensus-defined parameters in routine clinical documentation during a two-year period (2020–2021). The resulting dataset offers an initial step toward establishing structured prospective surveillance and may support future work to better understand and reduce CVAD-associated risks.

## Methods

### Study design and patient cohort

This mixed-methods study combined an interdisciplinary two-stage eDelphi process with a retrospective single-center analysis of CVADs used in pediatric oncology patients at a tertiary care institution in Lower Saxony, Germany. The study aimed to define clinically relevant parameters for structured CVAD surveillance and to examine how reliably these parameters can be captured within existing clinical documentation systems. The retrospective cohort included all patients aged 0–18 years who received a new cancer diagnosis at our hospital in 2020 or 2021 and underwent implantation of a CVAD during their treatment course. For the purpose of this analysis, CVADs were limited to TIVADs and TC-CVCs; other CVADs such as peripherally inserted central catheters (PICCs), or centrally inserted central catheters (CICCs) were not included. Patients were excluded if they were not followed at our institution after device implantation or if documentation of CVAD use and complications was incomplete.

Each implanted device was considered individually, including multiple devices per patient where applicable. For all device-based analyses (device type, age distribution, duration, complications, and removals), only the first CVAD implanted per patient (*n* = 112) was used to ensure comparability across clinical contexts. Additional devices (*n* = 14) represent subsequent implantations of a TIVAD or TC-CVC in the same patient and were included only in the descriptive total count. Not all catheter-related complications resulted in device removal, and not all removals were followed by implantation of another TIVAD or TC-CVC, as subsequent vascular access strategies were individualized based on the clinical situation and remaining treatment duration, including the use of alternative access types not covered by this analysis (e.g., PICCs or CICCs). The study was approved by the local Ethics Committee (No. 11396_BO_K_2024) and conducted in accordance with the Declaration of Helsinki [13].

### CVAD implantation and maintenance practices

All CVADs in pediatric oncology patients were inserted by pediatric surgeons according to a locally applicable standard operating procedure (SOP) defining the methodological steps and hygienic conditions of catheter placement in line with established technical and hygienic standards. CVAD implantation was performed under maximal barrier precautions in the pediatric surgery operating room. This included sterile gowns, caps, masks, and sterile gloves worn by all personnel, sterile draping of the patient, and the exclusive use of sterile instruments. Sterile, prepackaged catheter sets were opened only after vessel identification. Prior to insertion, catheters were flushed with 0.9% sodium chloride solution (NaCl 0.9%) to ensure patency and remove air. All devices were implanted using an open surgical approach, including skin incision, vessel preparation, venotomy, and catheter insertion. For TIVADs, a subcutaneous pocket was created for the port chamber, which was subsequently connected to the catheter. For TC-CVCs, subcutaneous tunneling was performed toward the cervical exit site. After completion of device implantation, aspiration was performed to confirm blood return, followed by immediate flushing with NaCl 0.9%. Catheters were locked postoperatively with heparin (100 IU/mL). Device batch numbers were documented in the operative report and assigned to the respective patient. Due to the open surgical technique, ultrasound guidance was not used during catheter placement. Catheter tip position was routinely confirmed postoperatively by chest radiography. During the study period, no specific institutional ‘central line-associated bloodstream infection (CLABSI) prevention campaigns’ or interventional programs were implemented beyond routine participation in a hospital-wide hand hygiene campaign. In addition, structured skills-based training programs, such as SICKO (’Sicherheit in der Kinderonkologie’ - ’Safety in Pediatric Oncology’), provide a practical framework for reinforcing safe handling practices in pediatric oncology. SICKO is a multidisciplinary training program that includes simulation-based skills training with a focus on CVAD handling, hygienic procedures, and team-based workflows. These training sessions are conducted repeatedly throughout the year with the pediatric oncology team and involve multiple professional groups, supporting interdisciplinary learning and shared safety practices [14]. Routine catheter maintenance followed local nursing standards. The injection site was inspected daily for signs of local inflammation, including swelling, redness, or tenderness. In cooperative patients, inspection and palpation were considered sufficient. In patients unable to cooperate, the dressing was changed daily if the injection site was not visible. Dressings were always replaced if they were loose, damp, or visibly soiled. Hand hygiene was strictly performed before and after any contact with the catheter or injection site. Dressing changes and catheter handling followed an aseptic no-touch technique. During dressing changes, the injection site was disinfected using a skin disinfectant. Before intravenous administration (after removal of the cap) at injection sites and when reconnecting catheter components, all connections were spray-disinfected using an alcohol-based skin disinfectant, unless disinfectant caps were used. After blood sampling and after medication administration, CVADs were flushed with NaCl 0.9%. Upon completion of use, each lumen of TC-CVCs and the port chamber of TIVADs were locked with 10 mL of NaCl 0.9% to maintain patency and prevent drug incompatibilities. For port systems, only syringes with a volume of ≥ 10 mL were used to avoid excessive pressure and potential device damage.

### eDelphi process, data extraction, and outcome definitions

To identify essential surveillance parameters, an interdisciplinary eDelphi process was conducted among experts from pediatric oncology, pediatric surgery, radiology, anesthesiology, microbiology, nursing, and quality management. In the first round, participants provided three free-text suggestions on parameters relevant to describing CVAD use and complications. In the second round, the consolidated items were rated on a binary scale (“relevant” or “irrelevant”), applying a predefined ≥ 80% agreement threshold for consensus [15]. Retrospective data extraction was performed manually from multiple digital clinical information systems: SAP IS-H (SAP SE, Walldorf, Germany) was used to obtain demographic data, diagnoses, procedures, and hospital stays. ALIDA, the digital long-term documentation archive at our institution, served as the primary source for operative reports, medical letters, treatment curves, and microbiological findings. The clinical documentation system m.life (medisite GmbH, Hannover, Germany) was used selectively to clarify missing entries. To ensure accuracy of manually extracted data, regular plausibility checks were performed during the extraction process, including verification of implantation, first infection, and explantation dates.

Catheter-related infection was defined as an infectious episode during CVAD use that met at least one of the following criteria: (i) explicit documentation of a catheter-associated infection in the medical record; (ii) catheter-directed anti-infective management, such as catheter lock therapy or targeted antibiotic treatment for a suspected catheter focus; and/or (iii) microbiological growth from the catheter tip after device removal. First infection requiring antibiotic therapy was defined as the first documented episode of fever during neutropenia in patients with a CVAD that led to the initiation of systemic intravenous antibiotics, in accordance with institutional protocols. Antibiotic administration therefore reflects empiric treatment for fever during neutropenia and should not be interpreted as evidence of confirmed catheter-related infection.

First positive blood culture was defined as the first documented blood culture yielding bacterial growth obtained from the CVAD during catheter use, irrespective of subsequent clinical interpretation or treatment decision. This endpoint captures microbiological events, including cultures later judged as contamination. The classification of a positive blood culture as a true infection or as contamination without clinical consequences (e.g., no prolonged antibiotic therapy or catheter removal) was determined by the clinically responsible physician. This decision was based on the spectrum of microorganisms, time to positivity, assessment by a clinical microbiologist, and the patient’s clinical condition. These assessments were made on an individual case basis and were not guided by a formal standard operating procedure. In addition, detailed device-level characteristics were extracted when available, including catheter type, number of lumens, caliber, material, and presence or absence of a cuff. These device specifications are summarized in Supplemental Table 1.

### Statistical analysis

All data were pseudonymized before analysis. The final dataset comprised demographic and clinical variables, device-related characteristics, and the full set of parameters defined through the eDelphi process together with one additional variable specified by the study team. All variables included in the final dataset were analyzed descriptively using RStudio Version 4.4.3 (Posit Software, Boston, Massachusetts, USA), including calculation of frequencies, medians, ranges, and graphical visualization of selected distributions. In addition, selected exploratory inferential analyses were performed to compare groups. Due to non-normal distributions, continuous variables were compared using the Mann-Whitney U test (MWU), and categorical variables were compared using Fisher’s exact test. A p-value < 0.05 was considered statistically significant.

## Results

### eDelphi-derived consensus surveillance parameters

In the eDelphi process, 29 experts from all disciplines involved in CVAD management were invited to participate; 13 contributed in the first and eight in the second round (response rates 44.8% and 27.6%). Based on the 39 parameters proposed in the first round, 26 items reached the predefined consensus threshold of ≥ 80% and were included as eDelphi-derived surveillance variables. Four additional parameters that did not reach the predefined consensus threshold (75% agreement) were retained due to their clinical relevance as judged by the study team. Of the 30 parameters identified through the eDelphi process, seven parameters were not available from routine medical data due to missing or inconsistent documentation. To improve the completeness of the dataset, one additional parameter was defined by the study team: the reason for changing a CVAD system (Table 1). Manual data extraction required approximately 40 min per patient file (range 20–50 min).

**Table 1:** Consensus-based CVAD surveillance parameters derived from the interdisciplinary eDelphi process

No.	Domain	Parameter	Consensus (%)	Inclusion decision	Routinely available
1	Outcomes	Catheter dwell time	100	Included	yes
2	Care	Catheter usage duration	75	Included by team	yes
3	Patient	Age and height	88	Included	yes
4	Outcomes	Dysfunction	88	Included	yes
5	Patient	Diagnosis	75	Included by team	yes
6	Device	Catheter type	88	Included	yes
7	Outcomes	Premature removal	100	Included	yes
8	Outcomes	Infection	100	Included	yes
9	Outcomes	Documentation of infection	100	Included	yes
10	Outcomes	Time to first infection	100	Included	yes
11	Outcomes	Positive blood culture	100	Included	yes
12	Outcomes	Bacteremia	100	Included	yes
13	Outcomes	Treatment of infection	100	Included	yes
14	Patient	Immune status	88	Included	yes
15	Patient	Stem cell transplantation	88	Included	yes
16	Patient	Type of therapy	88	Included	yes
17	Care	Handling of catheter (parents or nurses)	100	Included	no
18	Care	Used medication	100	Included	no
19	Patient	Reason for catheter implantation	88	Included	yes
20	Care	Nursing resources required for management	88	Included	no
21	Device	Length of catheter after implantation	88	Included	no
22	Device	Dislocation/area of implantation	100	Included	yes
23	Device	More than one lumen needed	100	Included	no
24	Device	Vein of implantation	75	Included by team	yes
25	Outcomes	Thrombosis	100	Included	yes
26	Device	Complications during operation	88	Included	yes
27	Device	Reoperation	100	Included	yes
28	Outcomes	Extravasation	100	Included	yes
29	Outcomes	Child discomfort	88	Included	no
30	Patient	Preference of parents	75	Included by team	no
31	Outcomes	Reason for change of CVAD system	NA	Included by team	no

The table summarizes the surveillance parameters identified through the interdisciplinary eDelphi process, including the level of agreement among participating experts (n = 8). Parameters are grouped into four domains (patient, device, care, and outcomes) reflecting different aspects of CVAD surveillance in pediatric oncology. Parameters were categorized as ’included’ when consensus (≥ 80% agreement) was achieved, or as ‘included by team’ when they did not reach the predefined consensus threshold but were considered clinically relevant by the study team. Item No. 31 was not suggested by the experts but was considered important by the study team and therefore included. Parameters that did not achieve consensus and were not considered important by the study team were not included and are therefore not shown

*CVAD* Central venous access device

## Study cohort and catheter characteristics

**Fig. 1:**
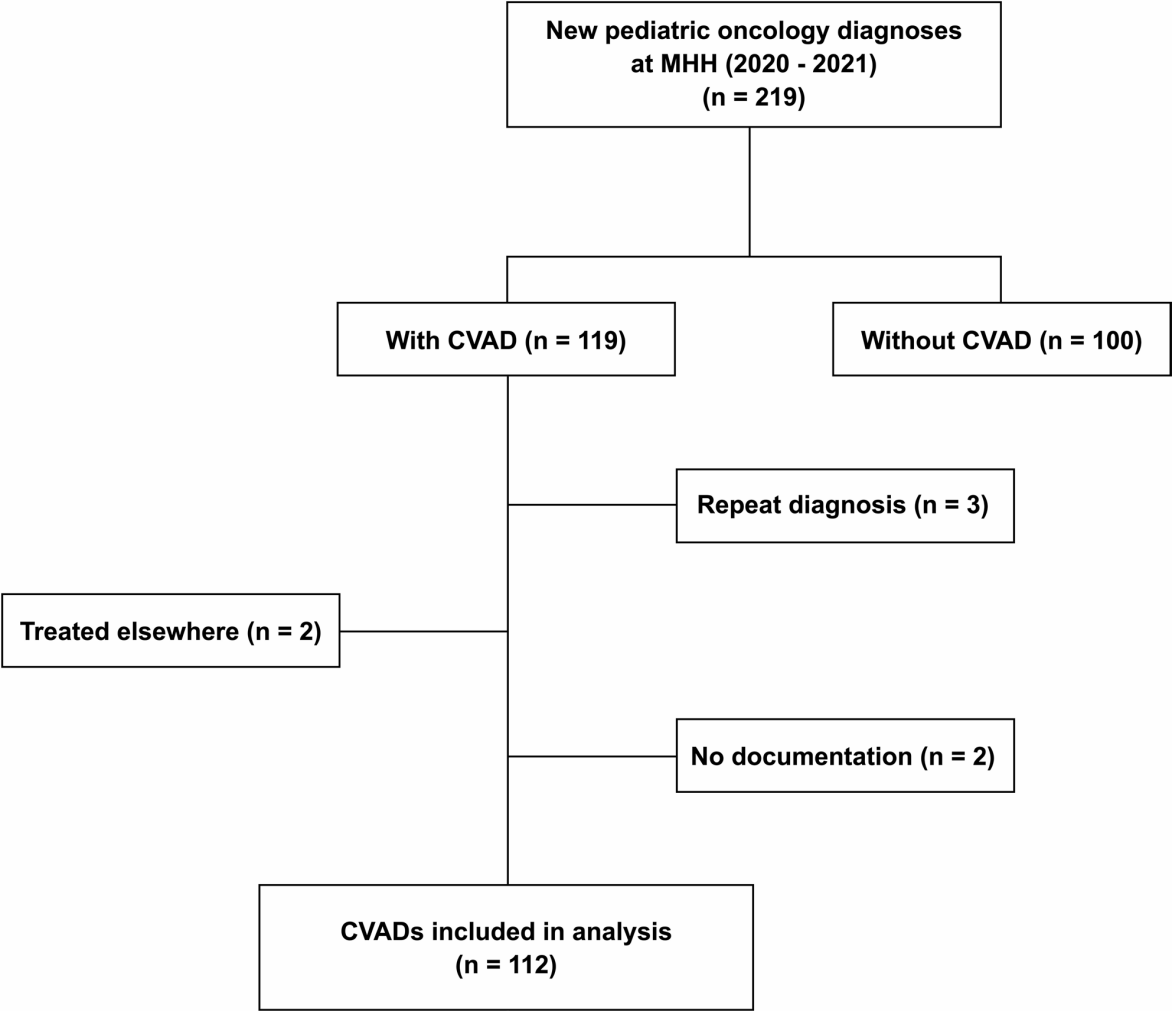
Patient inclusion flowchart. The figure illustrates the patient selection process for inclusion in the study (*n* = 112). For reasons of comparability, when more than one catheter was implanted in a single patient, only the first implanted catheter was included in the analysis. CVAD = central venous access device; MHH = Hannover Medical School

**Fig. 2:**
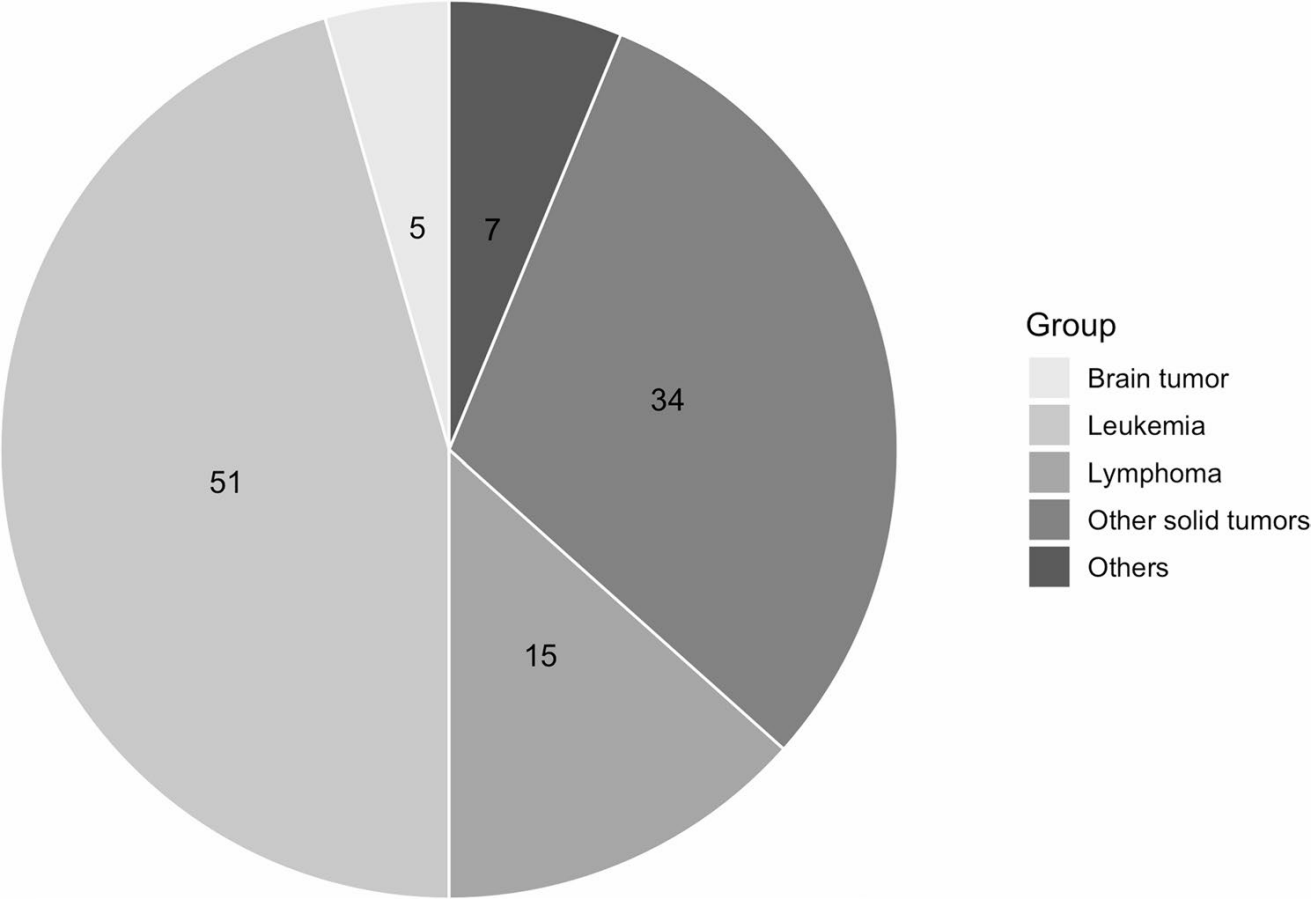
Distribution of primary oncological diagnoses. Patients were categorized into five groups according to their primary oncological diagnosis. Leukemias included acute lymphoblastic leukemia (n = 42) and acute myeloid leukemia (n = 9). Solid tumors were subdivided into brain tumors and other solid tumors. Additional categories comprised lymphomas and other diagnoses

A total of 219 pediatric patients received a cancer diagnosis during 2020–2021. Among these, 119 patients had a CVAD implanted, and 112 patients with 126 CVADs met all predefined inclusion criteria for retrospective analysis (64 female, 48 male). Seven cases were excluded because clinical documentation was incomplete or follow-up occurred outside our hospital (Fig. 1). CVAD implantation occurred a median of 7 days after diagnosis (range: 0–294). Patient demographics and catheter-related characteristics are summarized in Table 2. The distribution of underlying malignancies is shown in Fig. 2, with leukemias representing the largest diagnostic group (45.5%), followed by solid tumors and lymphomas.

**Table 2:** Overview of the study cohort

Characteristic	*N* / value
Number of patients	112
Median age (range)	6 years (0–18)
Sex (%)	Female: 64 (57.1) Male: 48 (42.9)
Total number of CVADs	126
TIVADs (including multiple per patient)	89 (99)
TC-CVCs (including multiple per patient)	23 (27)
Patients with > 1 CVAD (%)	13 (11.6)
Median time from diagnosis to CVAD implantation (range)	7 days (0–294)
Stem cell transplantations (autologous/allogeneic)	13 (7/6)

*CVAD* Central venous access device, *TC-CVC* Tunneled cuffed central venous catheter, *TIVAD* Totally implanted venous access device

Among the 112 first CVADs, 89 (79.5%) were TIVADs and 23 (20.5%) were TC-CVCs. Venous access sites differed by catheter type; TIVADs were most commonly inserted via the right cephalic vein, while TC-CVCs were most frequently placed via the right external jugular vein. The age distribution at the time of CVAD implantation covered the full pediatric spectrum, ranging from 0 to 223 months. Median age at implantation was 69 months for TC-CVCs and 72 months for TIVADs. Both catheter types showed a broad age distribution, with a wider spread observed among patients receiving TIVADs, including individual implantations in late adolescence (Supplemental Fig. 1).

Across all 126 devices, 33.729 catheter days were documented; 30.398 catheter days corresponded to the first 112 CVADs. Duration of use could be determined for 99 of the 112 primary CVADs, while 13 devices lacked complete duration information due to missing explantation dates or death with the device in situ. Median dwell time did not differ significantly between device types (MWU, *p* = 0.1726), with TIVADs remaining in place for a median of 293 days (range: 28–934) and TC-CVCs for 253 days (range: 49–546) (Supplemental Fig. 2). A total of 13 patients (11.6%) underwent hematopoietic stem cell transplantation, including seven autologous and six allogeneic procedures. The median interval between CVAD implantation and transplantation was 180 days (range 54–266).

### Complications and catheter removal

Complications are presented as secondary outcomes to contextualize outcome-related surveillance parameters and to illustrate their clinical relevance. Infectious and non-infectious complications occurred in both catheter types and were documented in 26 of the 112 first implanted devices (23.2%). An overview of all documented complications, including device-level characteristics such as catheter type and caliber, is provided in Supplemental Table 2. Across all first CVADs, 93 patients received intravenous antibiotics for fever during neutropenia (TC-CVCs, *n* = 20; TIVADs, *n* = 73). Among these, 49 patients had no pathogen detected in blood cultures, consistent with culture-negative febrile neutropenia. In addition, 44 positive blood cultures were documented (TC-CVCs, *n *= 12; TIVADs, *n *= 32), representing 21 distinct organisms. The most frequently identified pathogens were Escherichia coli, Staphylococcus epidermidis, Enterobacter cloacae, and Klebsiella pneumoniae. Based on the predefined criteria, 15 episodes were classified as catheter-related infections. Two infection-related timepoints were analyzed: the first clinically documented infectious episode requiring intravenous antibiotics and the first positive blood culture. The median time to first clinically documented infection was 30 days for TIVADs and 46 days for TC-CVCs, while the median time to first positive blood culture was 55 and 62 days, respectively (Supplemental Fig. 3a + b). Non-infectious complications included thrombosis (*n* = 3), catheter occlusion (*n* = 1), wound healing disorder (*n* = 4), mechanical dislocation (*n* = 3), and accidental catheter removal (*n* = 1).

A total of 21 devices (18.8%) were removed before completion of therapy. Early removals involved 12 of 89 TIVADs (13.5%) and 9 of 23 TC-CVCs (39.1%); this difference was statistically significant (Fisher’s exact test, *p* = 0.013). Infection was the most frequently documented reason for explantation (eight TIVADs and five TC-CVCs). Non-infectious complications that led to premature catheter removal were documented in eight cases. These included three mechanical dislocations, three wound healing disorders, one occlusion, and one accidental catheter removal.

## Discussion

Our study combined an interdisciplinary eDelphi consensus as its primary component with a retrospective data analysis to assess CVAD surveillance in pediatric oncology. We defined 30 key surveillance parameters through expert consensus but found only 23 could be extracted from existing records. While non-standardized and fragmented documentation of CVAD-related complications is a well-recognized challenge [16], the novelty of this study lies in the systematic linkage of expert-defined surveillance requirements with real-world documentation practice. Our intention was not to replace existing international frameworks, but to operationalize them for pediatric oncology practice by quantifying both the availability of consensus-derived parameters and the time burden required for manual data extraction, thereby explicitly demonstrating the gap between recommended surveillance requirements and routine data availability. Importantly, manual data abstraction from multiple systems took ∼40 min per patient file. This substantial time burden and data fragmentation represent a critical barrier to routine surveillance and benchmarking of CVAD-related outcomes.

By engaging an interdisciplinary expert panel for the eDelphi process, we achieved consensus on 26 key CVAD parameters to record for routine surveillance (≥ 80% agreement). Four additional items reaching only 75% consensus were still included in the analysis because they were unanimously deemed important by the study team. The broad range of perspectives helped ensure the parameters are practically relevant across specialties. The anonymous format reduced hierarchy bias, and the interdisciplinary composition supports the validity of the agreed-upon parameters [17, 18].

The outcome was a set of surveillance parameters capturing key domains of CVAD care, including patient/treatment characteristics, device details, insertion technique, line maintenance, and complication outcomes. These parameters are in line with what international experts have identified as essential for a vascular access minimum dataset. For example, the German Commission for Hospital Hygiene and Infection Prevention (KRINKO) and the Italian Association of Pediatric Hematology and Oncology (AIEOP) similarly emphasize tracking of infections, dwell time, and venous access site, among other factors [19, 20]. Likewise, a recent global Delphi study derived standardized items for vascular access reporting across domains from patient demographics to complications, reflecting priorities that strongly overlap with our eDelphi results [12]. This convergence suggests that our consensus-defined metrics are both evidence-based and broadly applicable, enhancing their compatibility with international surveillance efforts. Beyond infection-focused guidelines, our findings also align with broader international initiatives in vascular access care that emphasize standardized terminology, structured documentation, and interdisciplinary surveillance. Organizations such as the World Congress on Vascular Access (WoCoVA) [6], the Italian Group of Venous Access Devices (GAVeCeLT) [21], and the Infusion Nurses Society (INS) [22] have consistently advocated for harmonized vascular access reporting frameworks that extend beyond infectious outcomes to include device characteristics, insertion context, and longitudinal access management. In this context, the eDelphi-derived parameter set presented in our study complements these international efforts by providing a pragmatic, consensus-based framework tailored to pediatric oncology.

Some additional parameters (e.g. psychosocial factors, family preferences) extend beyond standard datasets to reflect specific needs in pediatric oncology. However, we found that seven of the expert-recommended parameters were largely missing or hard to find in routine records (e.g. the externally visible length of TC-CVC after implantation, patient-reported discomfort during catheter use, and medications administered via CVAD). This gap between expert consensus and practice highlights the need for standardized documentation so that these relevant factors can be captured prospectively. In this context, our findings illustrate that while a consensus-based surveillance dataset can be clearly defined, its full implementation is currently constrained by the scope and structure of routinely available clinical documentation. Overall, the eDelphi process confirmed that a structured documentation framework is both feasible and aligned with evidence-based care, providing a foundation for consistent multicenter surveillance of CVAD use.

### CVAD-related complications and the importance of a structured surveillance

Implanted CVADs are indispensable for delivering intensive therapy, but as expected, they incur risks of infection (CLABSI) and other non-infectious complications [23]. The reported complication rates should be interpreted as secondary outcomes and primarily serve to contextualize the relevance and feasibility of structured CVAD surveillance. In practice, the influence of individual factors like implantation timing and insertion technique is difficult to isolate in heterogeneous pediatric oncology cohorts. In our overall cohort, CVAD implantation occurred early after cancer diagnosis (median 7 days). Against this background, a focused assessment of implantation timing is limited by the wide variation in underlying diagnoses and treatment intensities. However, data from more homogeneous cohorts, such as children with acute lymphoblastic leukemia, suggest that early CVAD placement at diagnosis or during induction therapy is not associated with increased rates of infection or thrombosis [24].

The impact of ultrasound guidance on insertion-related complications depends on catheter type and insertion technique. Ultrasound-guided insertion has been shown to improve success rates and reduce the number of attempts during percutaneous central venous catheter placement in pediatric patients [25]. For TIVADs, evidence is mixed and largely derived from heterogeneous cohorts that are not limited to pediatric populations. One meta-analysis demonstrated superiority of open cephalic vein cut-down over closed subclavian cannulation with respect to pneumothorax and recommended open cut-down as the first-line approach, with ultrasound-guided closed cannulation as a second-line option [26]. In contrast, another meta-analysis reported higher primary implantation success rates for percutaneous subclavian puncture, with no significant differences in infectious or thrombotic complications, while pneumothorax occurred exclusively after percutaneous insertion [27]. In our institution, CVADs were implanted using a standardized open surgical technique with direct vessel visualization. It should be noted that procedural details commonly used to assess insertion success, such as the number of cannulation attempts, were not routinely documented and therefore could not be assessed in this retrospective analysis.

Complications were documented in 26 of the 112 first implanted devices (23.2%). This is in line with findings from another single-center study in pediatric oncology [3]. A recent meta-analysis, however, reported complication rates of up to 31% across pooled cohorts [23]. The slightly lower rate observed in our study may reflect the single-center design and differences in how complications were identified and documented. In our cohort, 21 patients (18.8%) experienced premature removal of the first CVAD due to complications, which is in line with findings from a long-term prospective pediatric oncology study from 2024 [28], but markedly lower than the 37% reported by van den Bosch et al. [29]. However, their cohort included not only TIVADs and TC-CVCs, but also non-tunneled and peripherally inserted devices, which may be removed more readily due to less invasive placement techniques and different clinical thresholds. Removal rates differed by catheter type: 39.1% of TC-CVCs were explanted early versus 13.5% of TIVADs. The majority (61.9%) of these early removals occurred in the context of infection, highlighting suspected or clinically associated CLABSI as a major driver of catheter loss. Our findings mirror prior studies showing higher infection and explantation rates for TC-CVCs compared to TIVADs [3, 30, 31]. In our series, we documented 44 positive blood cultures yielding 21 distinct pathogens (most commonly Escherichia coli, Staphylococcus epidermidis, and Klebsiella pneumoniae), consistent with the microbial diversity typically reported in pediatric CLABSI, including both gram-negative and gram-positive organisms [30, 32]. Non-infectious complications that led to premature catheter removal were documented in eight cases. This distribution is consistent with other studies reporting the spectrum of non-infectious CVAD-related complications in pediatric oncology [3].

Targeted measures have the potential to reduce catheter-associated complications [10], but effective implementation requires active surveillance. Current surveillance practices for CVAD performance probably fail to capture the full extent of the burden on pediatric patients and the healthcare system [9]. A first step is to implement structured prospective monitoring using the parameters identified by the eDelphi process. Embedding a standardized CVAD data form or checklist into the electronic health record could ensure that each device’s insertion, usage, and complications are uniformly captured. Routine surveillance programs should be established to monitor compliance and outcomes. Notably, the introduction of a specialized CVAD rounding team has proven effective in reducing complications. For example, Hanaki et al. reported that after initiating weekly multidisciplinary line rounds, the incidence of dislodgements dropped from 28.6% to 0%, and local infection rates fell from 17.9% to 2.6% [10]. This substantial improvement illustrates how focused surveillance and maintenance can directly translate into better patient outcomes. However, our analysis highlights how difficult this is to achieve in practice: relevant CVAD data were scattered across three separate CIS, often inconsistently recorded or only available in poorly legible handwritten form or as insufficiently scanned documents. This fragmentation not only limits retrospective analysis but likely results in an underestimation of the true complication burden. Manually extracting a complete catheter history took an average of 40 min per patient and involved repeated cross-checking. Frequently cited parameters such as the medications administered, the nursing workload, and the external catheter length after placement could not be evaluated. Modern catheter surveillance therefore requires more than just technical access; it depends on standardized, consensus-driven data structures and, ideally, smart systems that can support automated extraction and analysis [33]. Beyond technical infrastructure, effective CVAD surveillance also requires dedicated human resources. Even with centralized and standardized electronic documentation systems, trained clinical or research personnel are needed to ensure data completeness, perform quality checks, and maintain longitudinal surveillance. Our finding of a substantial manual data extraction burden underscores that personnel capacity is a critical prerequisite for implementing sustainable surveillance frameworks in routine clinical practice.

Emerging studies suggest that artificial intelligence-enabled surveillance and prediction systems can accelerate the early detection of catheter-related infections, enhance patient safety through timely interventions, reduce clinical workload by automating risk stratification and alerting, and support more cost-effective resource allocation [34]. Our study contributes a clinically validated parameter set and a practical demonstration of the current structural and personnel-related challenges that must be addressed to realize such systems.

### Strength and limitations

The study’s strengths include an interdisciplinary eDelphi consensus process to define key CVAD usage parameters, engaging clinicians in a structured consensus to minimize bias. Moreover, combining retrospective data analysis with the eDelphi consensus helped align actual CVAD use with the selected documentation parameters. Data extraction was performed via a structured manual review of electronic medical records, with standardized data entry and plausibility checks after each batch of entries to ensure accuracy.

However, the retrospective single-center design may limit generalizability, as findings primarily reflect local practice structures. In addition, the study was based exclusively on written clinical documentation and did not include direct observation of implantation or nursing practices, such that process-related aspects could only be assessed through recorded data. Routine clinical documentation lacked standard fields for some relevant variables, resulting in missing data and data gaps. While catheter-related infections could be identified based on predefined criteria, the etiology and clinical severity of febrile episodes could not be reliably assessed retrospectively, limiting differentiation between uncomplicated febrile neutropenia and true clinical sepsis. Seven patients with incomplete follow-up documentation were excluded from analysis, potentially introducing selection bias. Manual data aggregation, while carefully performed, inherently carries risks of subjective interpretation or entry error, although this was mitigated by a detailed, stepwise extraction protocol and regular cross-checks. Further limitations relate to the eDelphi process. Participation decreased in the second Delphi round, with eight experts completing the final survey. In addition, four parameters did not reach the predefined consensus threshold of ≥ 80% agreement but were retained based on unanimous agreement within the study team. These items were included to ensure clinical completeness of the surveillance framework and were not intended to redefine expert consensus. Overall, these factors should be considered when interpreting the findings, underscoring the need for prospective, multicenter studies with standardized data collection in pediatric CVAD care.

## Conclusions

This study identified consensus-based CVAD surveillance parameters and evaluated their availability in routine documentation. While a majority of these variables could be retrospectively extracted, key parameters were inconsistently documented or missing. These findings highlight critical barriers to structured surveillance and underscore the need for standardized CVAD data integration into electronic health records. Effective surveillance could help reduce complications, improve patient safety, and ease clinical workload through more targeted and timely interventions, which in pediatric oncology may also extend to parents or caregivers involved in catheter care and home-based use.

## Supplementary Information

Supplementary Material 1.

Supplementary Material 2.

Supplementary Material 3.

Supplementary Material 4.

Supplementary Material 5.

## Data Availability

The datasets generated and analyzed during this study are not publicly available due to patient confidentiality regulations but are available from the corresponding author upon reasonable request.

## Abbreviations

AIEOP: Italian Association of Pediatric Hematology and Oncology
CICC: Centrally inserted central catheters
CIS: Clinical information systems
CLABSI: Central line-associated bloodstream infection
CVAD: Central venous access device
eDelphi: Electronic Delphi
GAVeCeLT: Italian Group of Venous Access Devices
INS: Infusion Nurses Society
KRINKO: German Commission for Hospital Hygiene and Infection Prevention
MWU: Mann-Whitney U test
NaCl 0.9%: 0.9% sodium chloride solution
PICC: Peripherally inserted central catheters
SOP: Standard operating procedure
TC-CVC: Tunneled cuffed central venous catheter
TIVAD: Totally implanted venous access devices
WoCoVA: World Congress on Vascular Access
