# Supplementary material for: Missing data, missed risks: complications and documentation gaps of central venous access devices in pediatric oncology
Source: BMC Pediatr. 2026 Feb 10;26:150. doi: 10.1186/s12887-026-06612-0 (PMC12930751; doi:10.1186/s12887-026-06612-0)
Supplement: Supplementary file 1 — Supplementary Material 1. [file 12887_2026_6612_MOESM1_ESM.docx]

**Supplemental TABLE 1.** Catheter specifics of n = 112 CVADs

| Catheter (incl. SKU) | N | Lumen/caliber | Material | Cuff |
| --- | --- | --- | --- | --- |
| Bard Broviac^®️^  #0600060CE* | 2 | 1 / 4.2 Fr (1.4mm) | Silicone | SureCuff™️ Tissue Ingrowth Cuff |
| Bard Broviac^®️^  #0600520CE | 1 | 1 / 4.2 Fr (1.4mm) | Silicone | SureCuff™️ Tissue Ingrowth Cuff |
| Bard Broviac^®️^  #0600100CE | 4 | 1 / 6.6 Fr (2.2mm) | Silicone | SureCuff™️ Tissue Ingrowth Cuff |
| 1x Broviac^®️^ (not further specified) | 1 | 1 / n.a. | n.a. | n.a. |
| Bard Hickman^®️^  #0600310CE* | 7 | 2 / 7 Fr (2.33mm) | Silicone | SureCuff™️ Tissue Ingrowth Cuff |
| Bard Hickman^®️^  #0600570CE | 1 | 2 / 7 Fr (2.33mm) | Silicone | SureCuff™️ Tissue Ingrowth Cuff |
| Bard Hickman^®️^  #0606460CE* | 5 | 3 / 10 Fr (3.33mm) | Silicone | SureCuff™️ Tissue Ingrowth Cuff |
| Bard Hickman^®️^  #0606560CE | 1 | 3 / 10 Fr (3.33mm) | Silicone | SureCuff™️ Tissue Ingrowth Cuff |
| Bard Hickman^®️^  #0600360CE | 1 | 3 / 12.5 Fr (4.17mm) | Silicone | SureCuff™️ Tissue Ingrowth Cuff |
| Braun Celsite^®️^ Babyport #04433742 | 7 | 1 / 4.5 Fr (1.5mm) | Polyurethane | No cuff |
| Bard Titan Low-Profile Port #0605490CE | 45 | 1 / 6 Fr (2.0mm) | Polyurethane | No cuff |
| Bard Titan Low-Profile Port #0605300CE | 29 | 1 / 8 Fr (2.7mm) | Polyurethane | No cuff |
| Port (not further specified) | 8 | n.a. | n.a. | No cuff |

Fr = French catheter scale (1 Fr = ^1^/_3_ mm outer diameter); n.a. = not available; SKU = stock keeping unit; TC-CVC = tunneled cuffed central venous catheter; TIVAD = totally implanted venous access device. Broviac^®️^ and Hickman^®️^ catheters are classified as TC-CVCs, whereas ports are classified as TIVADs. *This catheter is supplied without a percutaneous insertion kit.
