# Supplementary material for: Missing data, missed risks: complications and documentation gaps of central venous access devices in pediatric oncology
Source: BMC Pediatr. 2026 Feb 10;26:150. doi: 10.1186/s12887-026-06612-0 (PMC12930751; doi:10.1186/s12887-026-06612-0)
Supplement: Supplementary file 2 — Supplementary Material 2. [file 12887_2026_6612_MOESM2_ESM.docx]

**SUPPLEMENTAL TABLE 2.** Complications and premature removals of n = 112 CVADs

| Complication | n (%) | Catheter type (TC-CVC/TIVAD) | Incidence rate per 1.000 catheter days* | Catheter type specifics (incl. SKU) |
| --- | --- | --- | --- | --- |
| **Total complications leading to dysfunction** | 26 (23.2) | **(9/17)** | **0.86 (0.56 - 1.25)** |  |
| Catheter infections leading to dysfunction | 13 (11.6) | (5/8) | 0.43 (0.23 - 0.73) | 1x Bard Broviac^®️^ (6.6 Fr), 1 lumen #0600100CE  1x Broviac^®️^, 2 lumen (not further specified)  1x Bard Hickman^®️^ (7 Fr), 2 lumen #0600310CE  2x Hickman^®️^ (10 Fr), 3 lumen #0606460CE  1x Braun Celsite^®️^ Babyport (4.5 Fr) #04433742  5x Bard Titan Low-Profile Port (6 Fr) #0605490CE  1x Bard Titan Low-Profile Port (8 Fr) #0605300CE  1x Port (not further specified) |
| Wound healing disorders | 4 (3.6) | (0/4) | 0.13 (0.04 - 0.34) | 2x Bard Titan Low-Profile Port (6 Fr) #0605490CE  2x Bard Titan Low-Profile Port (8 Fr) #0605300CE |
| Thrombosis | 3 (2.7) | (0/3) | 0.09 (0.02 - 0.29) | 1x Bard Titan Low-Profile Port (6 Fr) #0605490CE  2x Bard Titan Low-Profile Port (8 Fr) #0605300CE |
| Dislocation | 3 (2.7) | (2/1) | 0.09 (0.02 - 0.29) | 1x Bard Broviac^®️^ (4.2 Fr), 1 lumen #0600060CE  1x Bard Hickman^®️^ (7 Fr), 2 lumen #0600310CE  1x Bard Titan Low-Profile Port (6 Fr) #0605490CE |
| Infection and thrombosis combined | 1 (0.9) | (0/1) | 0.03 (0.00 - 0.18) | 1x Bard Titan Low-Profile Port (6 Fr) #0605490CE |
| Accidental removal | 1 (0.9) | (1/0) | 0.03 (0.00 - 0.18) | 1x Bard Broviac^®️^ (6.6 Fr), 1 lumen #0600100CE |
| Occlusion | 1 (0.9) | (1/0) | 0.03 (0.00 - 0.18) | 1x Hickman^®️^ (10 Fr), 3 lumen #0606460CE |
|  |  |  |  |  |
| Catheter infections | 15 (13.4) | (6/9) | 0.49 (0.28 - 0.81) |  |
| At least one positive blood culture during follow-up | 44 (39.3) | (12/32) | 1.45 (1.05 - 1.94) |  |
|  |  |  |  |  |
| Planned removal after completion of therapy | 75 (67) | (12/63) | 2.47 (1.94 - 3.09) |  |
| Total premature removals | 21 (18.8) | (9/12) | 0.69 (0.43 - 1.06) |  |
| Premature removals due to infection | 13 (11.6) | (5/8) | 0.43 (0.23 - 0.73) |  |
| Wound healing disorder | 3 (2.7) | (0/3) | 0.09 (0.02 - 0.29) |  |
| Dislocation | 3 (2.8) | (2/1) | 0.09 (0.02 - 0.29) |  |
| Accidental removal | 1 (0.9) | (1/0) | 0.03 (0.00 - 0.18) |  |
| Occlusion | 1 (0.9) | (1/0) | 0.03 (0.00 - 0.18) |  |
|  |  |  |  |  |
| Death | 4 (3.6) | (2/2) | 0.13 (0.04 - 0.34) |  |
| Infection after ending of therapy | 1 (0.9) | (0/1) | 0.03 (0.00 - 0.18) |  |
| Planned change of catheter system | 2 (1.8) | (0/2) | 0.07 (0.01 - 0.24) |  |
| Explantation not documented | 8 (7.1) | (0/8) | 0.26 (0.11 - 0.52) |  |
| Therapy at another clinic | 1 (0.9) | (0/1) | 0.03 (0.00 - 0.18) |  |

*Incidence rate (lower 95% confidence interval - upper 95% confidence interval). CVAD = central venous access device; Fr = French catheter scale (1 Fr = ^1^/_3_ mm outer diameter); SKU = stock keeping unit; TC-CVC = tunneled cuffed central venous catheter; TIVAD = totally implanted venous access device. Broviac^®️^ and Hickman^®️^ catheters are classified as TC-CVCs, whereas ports are classified as TIVADs.
