# Supplementary figures and images for: Missing data, missed risks: complications and documentation gaps of central venous access devices in pediatric oncology
Source: BMC Pediatr. 2026 Feb 10;26:150. doi: 10.1186/s12887-026-06612-0 (PMC12930751; doi:10.1186/s12887-026-06612-0)

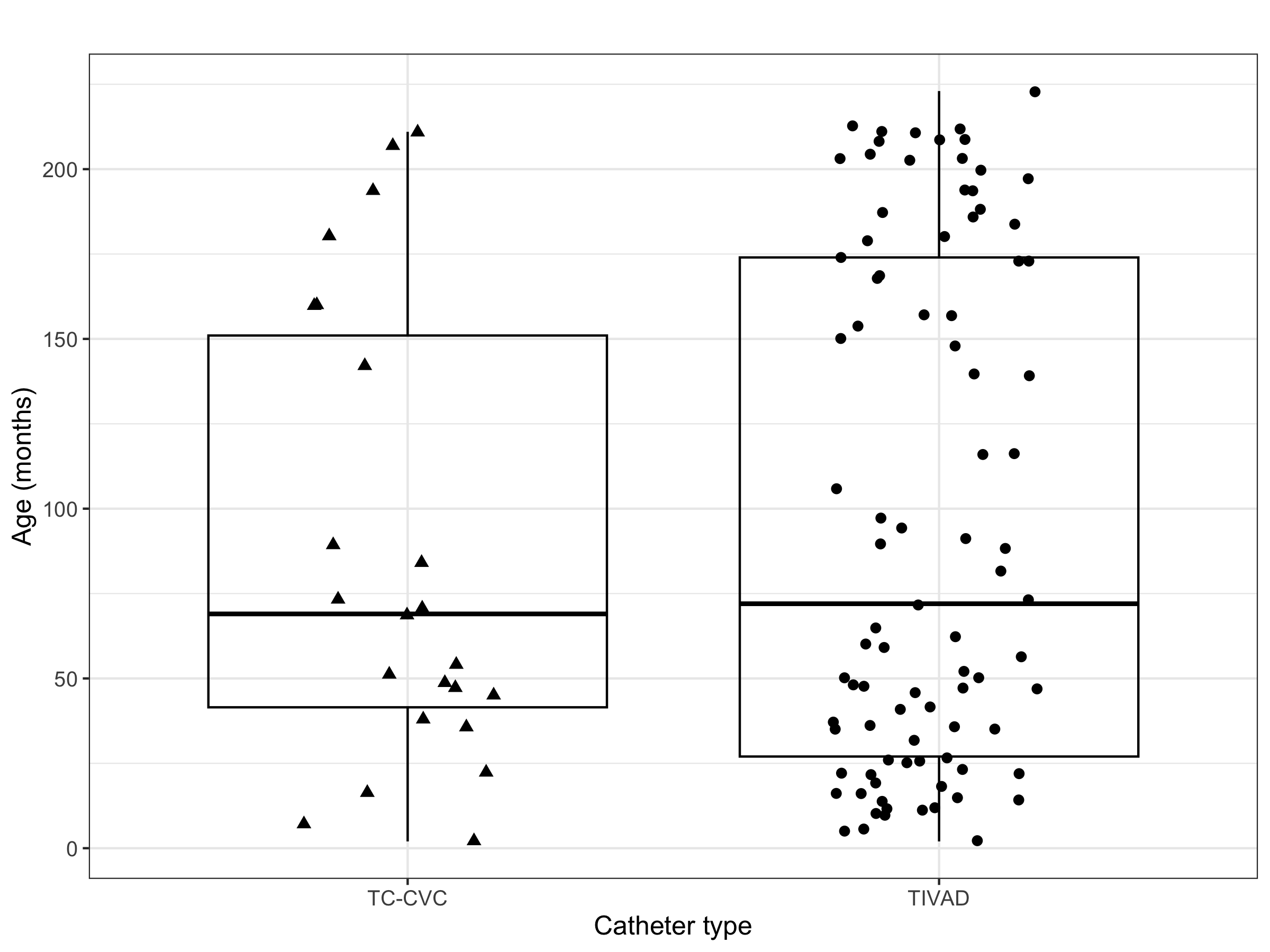

Supplement: Supplementary file 3 — Supplementary Material 3. [file 12887_2026_6612_MOESM3_ESM.tiff]

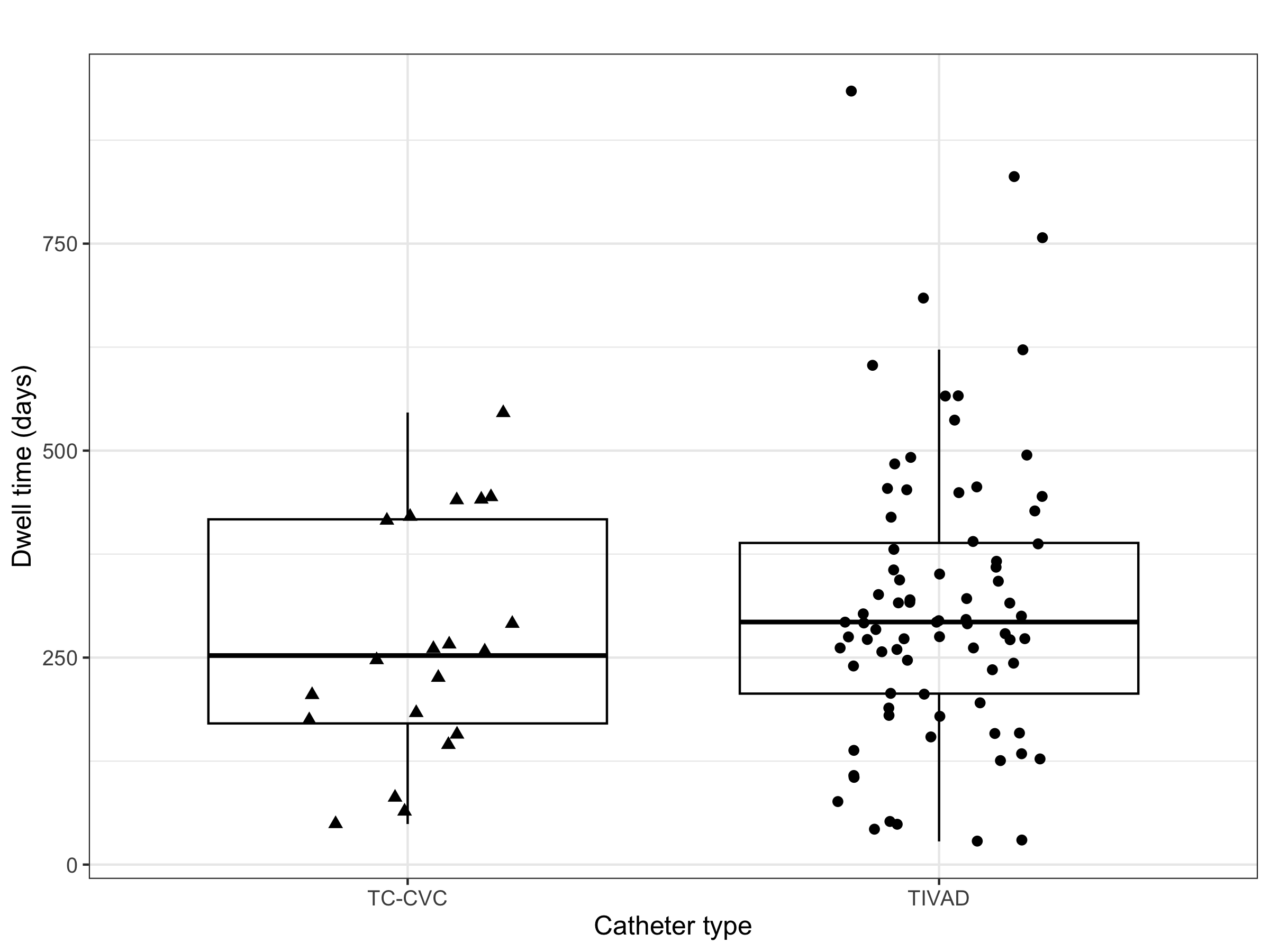

Supplement: Supplementary file 4 — Supplementary Material 4. [file 12887_2026_6612_MOESM4_ESM.tiff]
